# Antioxidant availability trades off with warning signals and toxin sequestration in the large milkweed bug (*Oncopeltus fasciatus*)

**DOI:** 10.1002/ece3.9971

**Published:** 2023-04-07

**Authors:** H. Cecilia Heyworth, Prayan Pokharel, Jonathan D. Blount, Christopher Mitchell, Georg Petschenka, Hannah M. Rowland

**Affiliations:** ^1^ Predators and Toxic Prey Research Group Max Planck Institute for Chemical Ecology Jena Germany; ^2^ Centre for Ecology and Conservation, College of Life and Environmental Sciences University of Exeter Exeter UK; ^3^ Department of Applied Entomology, Institute of Phytomedicine University of Hohenheim Stuttgart Germany

**Keywords:** aposematism, cardenolides, honest signaling, oxidative state, resource competition

## Abstract

In some aposematic species the conspicuousness of an individual's warning signal and the concentration of its chemical defense are positively correlated. Several mechanisms have been proposed to explain this phenomenon, including resource allocation trade‐offs where the same limiting resource is needed to produce both the warning signal and chemical defense. Here, the large milkweed bug (*Oncopeltus fasciatus*: Heteroptera, Lygaeinae) was used to test whether allocation of antioxidants, that can impart color, trade against their availability to prevent self‐damage caused by toxin sequestration. We investigated if (i) the sequestration of cardenolides is associated with costs in the form of changes in oxidative state; and (ii) oxidative state can affect the capacity of individuals to produce warning signals. We reared milkweed bugs on artificial diets with increasing quantities of cardenolides and examined how this affected signal quality (brightness and chroma) across different instars. We then related the expression of warning colors to the quantity of sequestered cardenolides and indicators of oxidative state—oxidative lipid damage (malondialdehyde), and two antioxidants: total superoxide dismutase and total glutathione. Bugs that sequestered more cardenolides had significantly lower levels of the antioxidant glutathione, and bugs with less total glutathione had less luminant orange warning signals and reduced chroma of their black patches compared to bugs with more glutathione. Bugs that sequestered more cardenolides also had reduced red–green chroma of their black patches that was unrelated to oxidative state. Our results give tentative support for a physiological cost of sequestration in milkweed bugs and a mechanistic link between antioxidant availability, sequestration, and warning signals.

## Graphical abstract

We raised milkweed bugs on artificial diets with increasing quantities of cardenolides and then examined how this affected oxidative state and signal quality (brightness and chroma) across different life stages. Glutathione decreased in all individuals with increasing levels of sequestered cardenolides. Bugs with more total glutathione had brighter warning signals but these signals were not related to sequestration.

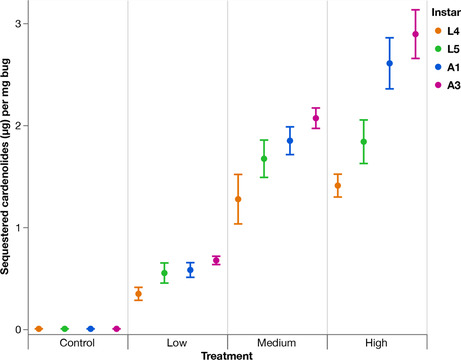

## 1 INTRODUCTION

The conspicuous colors of aposematic animals serve as important signals of chemical defenses (Sherratt, 2002; Wallace, 1889). In some aposematic animals warning signals and chemical defenses are positively correlated, both within species (Bezzerides et al., 2007; Blount et al., 2012; Maan & Cummings, 2012; Vidal‐Cordero et al., 2012) and across species (Cortesi & Cheney, 2010). Several mechanisms have been proposed to explain this phenomenon (Blount et al., 2009; Holen & Svennungsen, 2012; Lee et al., 2011). One mechanism that predicts a positive relationship between conspicuousness and defense in prey is resource allocation trade‐offs, where variable access to resources may result in differential costs of signaling (Blount et al., 2009; Holen & Svennungsen, 2012). One possible shared resource is energy, which can be limiting for the sequestration or biosynthesis of toxins (Holloway et al., 1991) and the expression of warning signals (Srygley, 2004). Another is antioxidants, which are relevant if sequestration of toxins imposes a metabolic cost in the form of oxidative stress (Ahmad, 1992; Blount et al., 2009; Tollrian & Harvell, 1999).

In Blount et al.'s (2009) resource competition framework, pigments that are used in prey warning signals play a role both in producing the signal and in preventing self‐damage when storing toxins (due to their antioxidant properties). When antioxidants are required to enable high levels of toxicity, then the most toxic species are predicted to be the most conspicuous because they gain access to more antioxidants (or are genetically disposed to produce more), than those that are less bright and less toxic (see figure 1 in Blount et al., 2023). There are many critiques of the idea that warning signals are costly, one of which is that it is unclear whether warning signals ‘use up’ resources that are needed to produce chemical defenses (Guilford & Dawkins, 1993). The potential influence of antioxidant availability and oxidative stress on the development of aposematic displays has received limited empirical attention (Ojala et al., 2005; Sandre et al., 2007), though Blount et al. (2023) found that monarch butterflies (*Danaus plexippus*) that sequestered higher concentrations of chemical defense experienced higher levels of oxidative damage, and monarchs with lower oxidative damage allocated more resources to color and toxicity than monarchs with higher oxidative damage. Because Blount et al. (2023) reared monarchs on different milkweed hostplants (*Apocynaceae*) to manipulate the amounts of toxins the butterflies sequestered, and because different hostplants vary in many traits (not just chemical defense), the costs that Blount et al. (2023) detect may not result only from varied sequestration. A study that varies chemical defense content while holding other traits constant could provide a clearer test for a mechanistic link between oxidative stress, warning colors, and sequestration of chemical defenses.

Here we test Blount et al.'s (2009) resource competition model using the large milkweed bug, *Oncopeltus fasciatus* (Hemiptera, Lygainae), a conspicuously patterned orange and black insect. *O. fasciatus* feed on seeds or seedpods of milkweed plants (*Asclepias* spp; Burdfield‐Steel & Shuker, 2014; Feir, 1974), which produce cardenolides (Brower, 1969; Roeske et al., 1976). *O. fasciatus* not only tolerates cardenolides, but also sequesters these toxins for their own defense in a specialized, vacuolated layer of cells beneath their outer layer of epidermis (Bramer et al., 2017; Duffey et al., 1978; Scudder & Meredith, 1982). *O. fasciatus* individuals vary in the amount and structure of the cardenolides they sequester when feeding on the same host species (Isman et al., 1977) and the intensity of their colouration also varies in the wild (Davis, 2009; Rodríguez‐Clark, 2004). The pigments determining colouration in *O. fasciatus* are pteridines such as xanthopterin, isoxanthopterin, and 2‐amino‐4‐hydroxypteridine, and pterins such as erythropterin (Bartel et al., 1958; Good & Johnson, 1949; Hudson et al., 1959). These pigments have the potential to function as biological antioxidants, though evidence for their redox behavior is sparse (Martínez & Barbosa, 2010; McGraw, 2005; Reibnegger, 2018).

Because directly controlling the level of antioxidant defense in an animal is challenging experimentally, we modulated the quantity of diet‐derived toxin available to the individual to test whether (1) the quantity of sequestered cardenolides by *O. fasciatus* is associated with changes in oxidative state; and (2) oxidative state affects the capacity of *O. fasciatus* to produce warning signals displays. We reared *O. fasciatus* on artificial diets with an increasing amount of cardenolides to modulate dietary toxins and measured sequestered cardenolides across different instars. To determine the costs of sequestration we measured three indicators of oxidative state: oxidative damage via lipid peroxidation (malondialdehyde (MDA)), and two components of antioxidant defense: total superoxide dismutase (SOD) and total glutathione. We measured individual signal quality (brightness and chroma) and related this to oxidative state.

There are a number of potential scenarios for co‐variation between antioxidants, pro‐oxidants, and the resulting oxidative stress (Costantini & Verhulst, 2009). When ingested, many plant allelochemicals can cause oxidative lipid damage (Ahmad, 1992). If this is the case for *O. fasciatus* we would predict a positive correlation between individual levels of cardenolides sequestered and oxidative lipid damage, or stable levels of lipid damage but decreases in antioxidant defenses, measured by SOD, and total glutathione content (GSH). If, as predicted by Blount et al.'s (2009) model, some individuals have greater access to antioxidants, then for a given level of access to toxin we would predict individuals that sequester more cardenolides to also invest more in warning signals, as well as a positive correlation between color and toxicity. By using a controlled artificial diet, and a model species that naturally varies in both signal and toxicity, we can rigorously test the assumptions of Blount et al.'s (2009) resource competition model, as well as contribute to the growing literature examining honest signaling in aposematic species.

## 2 MATERIALS AND METHODS

### 2.1 Insect rearing and artificial diet

*Oncopeltus fasciatus* were obtained from a long‐term laboratory colony (originally from the United States) maintained on sunflower seeds. *O. fasciatus* develop through five instars, from their first nymphal stage (L1) through L2, L3, and L4, to their fifth (L5), after which they molt into adults. We split our experiments into three batches for which insects were reared serially. We seeded each batch with third nymphal stage (L3) bugs, and in each batch divided 60–100 L3 from a breeding colony into four treatment groups of 15–25 individuals each.

We raised *O. fasciatus* on four diets: three with increasing amounts of added ouabain and digitoxin, and one as a control diet with no added toxins. These cardenolides were selected because they are available commercially, and because they mimic the natural spectrum of cardenolides available in the seeds (ouabain is one of the most polar cardenolides, and digitoxin is one of the most apolar). We followed Pokharel et al.'s (2021) method to prepare an artificial diet which consisted of sunflower seeds, wheat germ, casein, sucrose, Wesson's salt, vitamins, methyl 4‐hydroxybenzoate, sorbic acid, olive oil, and cardenolides (only for the treatment groups, not for controls), which were combined with Agar and provided in the lids of 2 mL Eppendorf tubes sealed with a piece of stretched parafilm to create an artificial seed. The control (C) diet had no cardenolides added, and Low (L), Medium (M), and High (H) diets had an added 2, 6, and 10 mg cardenolides (an equimolar mixture of digitoxin and ouabain; Sigma‐Aldrich, Taufkirchen, Germany; Figure S1) per g dry weight of diet. These three concentrations were chosen as they fall within the range of dietary toxins naturally present in milkweed seeds (*Asclepias* spp; Isman, 1977; Isman et al., 1977; Rubiano‐Buitrago et al., 2023). The treatment groups were reared in plastic boxes (15 × 11 × 5 cm) with water supplied in Eppendorf tubes plugged with dental cotton and two portions of the artificial diet that were replenished once per week.

### 2.2 Photography and color analysis

We checked the insect boxes daily to monitor the bugs' molting. We took photographs of *O. fasciatus* individuals at the approximate end of nymphal stages 4 and 5 and twice within the adult stage (recently molted adults A1, and adults 5 to 10 days after molting which we termed A3). A1 adults were photographed approximately 1 day after the imaginal molt, to allow the bright red colouration apparent in the first hours after molting to reach regular adult colouration. Individuals were photographed once for image analysis. We used a Nikon D7000 digital SLR camera (Nikon) and a UV‐Nikkor 105 mm f/4.5 s. The lens was fitted with a custom‐built ring illumination that illuminated the bugs with LEDs emitting light with a wavelength from 380 to 780 nm (Figure S2), and a filter changer that allowed switching between a Baader UV‐IR blocking filter (Baader Planetarium; permitting only visible spectrum light from 420 to 680 nm) and a Baader UV pass filter (permitting ultraviolet light from 320 to 380 nm). Approximately half of the individuals in each dietary treatment group at each instar were randomly selected for photography. We sedated individual insects using CO_2_ and photographed them with dorsal side facing upwards on a color palette (ColorChecker Passport Photo 2, X‐rite, Pantone) and alongside an identifying label and a 40% Spectralon gray standard (Labsphere Inc.). We took three pictures with increasing exposure times (0.2, 0.33, and 0.77) with an aperture of 1.3× for both filters (that is, six pictures per bug).

We analyzed the images using the micaToolbox (Troscianko & Stevens, 2015) in ImageJ software 1.51 (Rasband, 1997–2018). Because digital cameras often show a non‐linear relationship between the pixel value recorded and changes in light intensity, the images were first normalized (Stevens et al., 2007) and then linearized to a device‐independent sRGB. Because *O. fasciatus* reflect negligible amounts of UV we used only photographs in the visible spectrum and converted the sRGB values to L*a*b* color space (CIELAB 1976; Commission Internationale de l'Eclairage; http://cie.co.at; Luo, 2014). CIELAB color space represents color in triplet coordinates of lightness and hue that approximates the red–green and yellow–blue opponent channels of humans (Luo, 2014). In each photograph we delineated consistent indicative regions of interest. In the nymphs we measured the dorsal side of abdominal segments—one orange section was chosen to avoid black markings, and we also measured the right side short melanised wing pads. In the adults we measured the orange and black wing segments (see Figure S3). We used the micaToolbox to measure the red, green, and blue, and L, A, and B values for each photograph and patch.

### 2.3 Homogenization of samples

After photography the bugs were placed into labeled Eppendorf tubes, weighed, flash‐frozen in liquid nitrogen, and stored at −80°C. Due to their smaller size, L4 nymphs were pooled into groups of two to have enough material for chemical and oxidative state assays. Each sample was homogenized in a 1:20 ratio of PBS buffer solution (pH 6.6, 50 mM, with 1 mM EDTA) to bug body mass using a FastPrep homogenizer (MP Biomedicals, LLC, US) at 10 m/s for 15 s. Tubes were centrifuged at 16,000 *g* and 4°C for 4 min, and the clear supernatant of the homogenate was transferred to a new 2 mL Eppendorf tube. Four aliquots were taken from each homogenate and placed into individual Eppendorf tubes. All aliquoting was done on ice. First, for the total glutathione (GSH) assay, 150 μL metaphosphoric acid (MPA) was added to 150 μL homogenate, vortexed, and left at room temperature for 5 min. The resulting mixture was centrifuged at 956 *g* and 4°C for 2 min, and the supernatant pipetted into a new 1.5 mL Eppendorf tube. Second, for the SOD assay, 50 μL homogenate was added to a new 1.5 mL tube with 50 μL sugar buffer (PBS with 12.6 mM mannitol and 4.2 mM sucrose) and vortexed. Third, for HPLC analysis, 100 μL of the homogenate was transferred to a new 1.5 mL Eppendorf tube. The fourth remaining 20 μL aliquot was used for MDA analysis. All samples were then frozen at −80°C.

### 2.4 Determination of oxidative stress and cardenolide concentration

We performed three oxidative state assays from the aliquoted homogenates: total glutathione (GSH), total SOD, and MDA. These assays were chosen to obtain a broad overview of biomarkers of oxidative state. GSH is an antioxidant molecule which serves as a nucleophilic co‐substrate to glutathione transferases in the detoxification of xenobiotics and is an essential electron donor to glutathione peroxidases in the reduction of hydroperoxides (Arias & Jakoby, 1976). SOD is a metalloenzyme that catalyzes the dismutation of superoxide into oxygen and hydrogen peroxide, forming a crucial part of intracellular antioxidant defenses (Malmström et al., 1975). MDA is formed by the β‐scission of peroxidised polyunsaturated fatty acids, and therefore is a definitive marker of oxidative lipid damage (Lapenna et al., 2001).

#### 2.4.1 Total glutathione (GSH)

Total GSH was assayed by measuring the enzymatic recycling of glutathione reductase for the quantification of GSH (Cayman Chemical, #703002). The homogenate was diluted (1:2 v/v) to fit the absorbance values within the range of the standard curve. Samples were assayed in duplicate, as per the kit instructions. Data are reported as nmol per mg of bug.

#### 2.4.2 Superoxide dismutase

Total SOD was assayed by measuring the dismutation of superoxide radicals generated by xanthine oxidase and hypoxanthine. We followed the instruction of the kits (Cayman Chemical #706002) except that we diluted the samples (1:50 v/v) to ensure that SOD activity fell within the range of the standard curve. Samples were assayed in duplicate and are reported as units of SOD activity (U) per mg of bug.

#### 2.4.3 Malondialdehyde

MDA was measured using HPLC with fluorescence detection (Agilent Technologies), using a modified version of Agarwal and Chase's method (Agarwal & Chase, 2002; Nussey et al., 2009). All chemicals were HPLC grade, and chemical solutions were prepared with ultra‐pure water (Milli‐Q Synthesis; Millipore). We transferred a 20 μL aliquot of each homogenized sample into 2 mL capacity polypropylene screw‐top microcentrifuge tubes and added 20 μL butylated hydroxytoluene (0.05% w/v in 95% ethanol), 40 μL 2‐thiobarbituric acid (TBA; 42 mM), and 160 μL phosphoric acid (H_3_PO_4_; 0.4 M). Samples were capped, vortexed for 2 s, and then heated at 100°C for 1 h in a dry bath incubator to allow formation of MDA‐TBA adducts. Samples were centrifuged for 1 min at 13,300 *g*, cooled for 5 min on ice before adding 160 μL *n*‐butanol to each tube and vortexing for 10 s. Tubes were centrifuged for 3 min at 12,000 *g* at 4°C, before the upper butanol phase was collected and transferred to an HPLC vial for analysis. Samples (20 μL) were injected into a HPLC system fitted with a Hypersil ODS C18 column (5 μm, 100 × 4.6 mm, HSA‐212‐510R, Fisher Scientific, USA). The mobile phase was methanol buffer (40:60 v/v), the buffer was an anhydrous solution of potassium monobasic phosphate (50 mM) at pH 6.8 (adjusted using 5 M potassium hydroxide solution), running isocratically over 3.5 min at a flow rate of 1 mL/min at 37°C. Data were collected using a fluorescence detector (RF2000; Dionex Corporation) set at 515 nm (excitation) and 553 nm (emission). For calibration a standard curve was prepared using serial dilutions of 5 μM 1,1,3,3‐tetraethoxypropane (which hydrolyses to produce MDA) in 40% ethanol. Data are presented as nmols MDA per mg of bug.

#### 2.4.4 Cardenolide analysis

To analyze the amount of sequestered cardenolides in the sample aliquot, we freeze‐dried the sample to remove the water content and vortexed the residue in 1 mL HPLC‐grade methanol containing 0.01 mg/mL of oleandrin (PhytoLab GmbH & Co. KG) as an internal standard. To facilitate dissolution of cardenolides, we immersed the sample in an ultrasonic bath for 30 min. After centrifugation at 16,100 *g* for 3 min, the supernatant was collected and the sample was extracted one more time with 1 mL of pure methanol. The supernatants were pooled and dried under a flow of nitrogen gas. We dissolved the remaining pellet in 100 μL methanol by agitating in the Fast Prep homogenizer and filtered into HPLC vials using Rotilabo syringe filters (nylon, pore size: 0.45 μm, diameter: 13 mm, Carl Roth GmbH & Co. KG). We injected 15 μL of sample into an Agilent HPLC (Agilent technologies) equipped with an EC 150/4.6 NUCLEODUR C18 Gravity column (3 μm, 150 mm × 4.6 mm, Macherey‐Nagel) and a photodiode array detector. Cardenolides were separated and eluted at a constant flow rate of 0.7 mL/min at 30°C using the following acetonitrile‐water gradient: 0–2 min 16% acetonitrile, 25 min 70% acetonitrile, 30 min 95% acetonitrile, 35 min 95% acetonitrile, 37 min 16% acetonitrile, 10 min reconditioning at 16% acetonitrile. Peaks with symmetrical absorption maxima between 218 and 222 nm were recorded as cardenolides (Malcolm & Zalucki, 1996). Finally, we estimated the amount of cardenolides per sample by comparing the ouabain peak area to the area of the internal standard, since digitoxin was not detected (Pokharel et al., 2021).

### 2.5 Statistical analysis

All analyses were performed in JMP Pro 16.2.0 statistical software (SAS Institute). We analyzed how treatment affected the quantity of cardenolides sequestered constructing a linear mixed model using restricted maximum likelihood and included treatment as a continuous variable; developmental stage, and the interaction between treatment and developmental stage as fixed effects; and batch as a random effect. We analyzed how sequestration affected oxidative state constructing a linear mixed model using restricted maximum likelihood; included sequestered cardenolide per bug, developmental stage, and the interaction between sequestered cardenolides and developmental stage as fixed effects; and batch as a random effect. We analyzed how sequestered cardenolides per bug and oxidative state affected signal quality (L* brightness, and hue a* and b*), constructing a linear mixed model using restricted maximum likelihood with developmental stage, oxidative stress markers, sequestered cardenolides, and the interaction between oxidative stress markers and sequestered cardenolides as fixed effects, and batch as a random effect. We fitted the data to the linear regression model using a smoothed conditional mean and plotted 95% confidence intervals.

## 3 RESULTS

### 3.1 Effect of diet on sequestration

**FIGURE 1:**
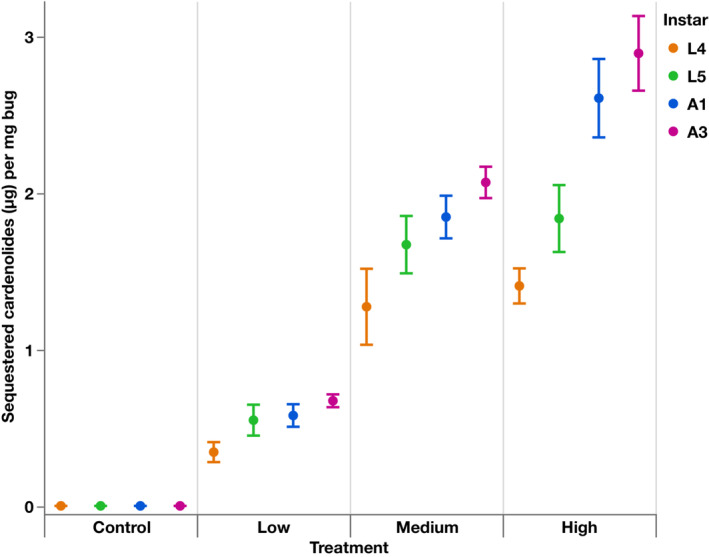
Mean concentration (±SE) of cardenolides sequestered (μg/mg) by nymphs L4 and L5, and by adults A1 and A3 of *O. fasciatus* individuals (*N* = 183) when raised on Control (no cardenolides), Low (2 mg/g), Medium (6 mg/g), and High (10 mg/g) cardenolide diets of roughly equimolar ouabain and digitoxin.

All *O. fasciatus* on experimental diets sequestered cardenolides, and all on the control diet had no cardenolides (Figure 1). The amount of cardenolides sequestered by individuals increased significantly with increasing cardenolide concentration in the diet (*F*_1,173.1_ = 662.88, *p* < .0001; estimate = 0.22 ± 0.009). There was a significant interaction between treatment and instar (*F*_3,173.1_ = 14.11, *p* < .0001) where the effects of diet and instar had different slopes (L5 estimate = −0.003 ± 0.01; A1 estimate = 0.047 ± 0.01; A3 estimate = 0.064 ± 0.01).

### 3.2 Cardenolide sequestration and oxidative stress

**FIGURE 2:**
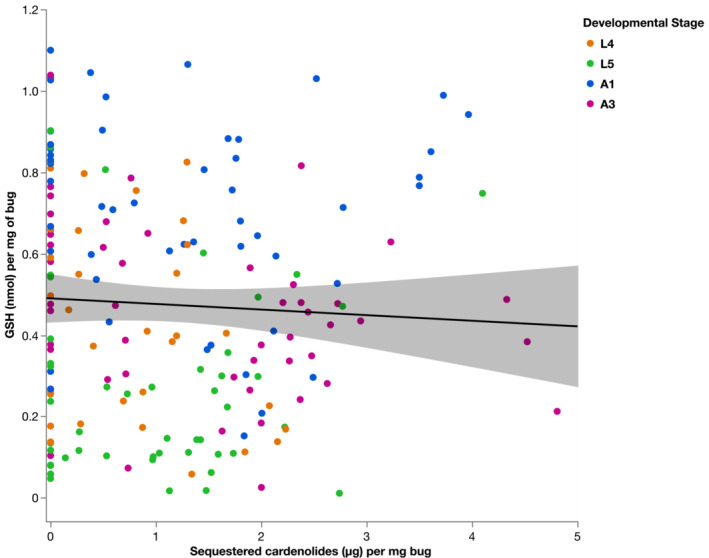
Correlation between sequestered cardenolides and total glutathione (GSH) in *O. fasciatus* individuals (*N* = 183). L4, L5 represent the nymphal stages Level 4 and 5. Adult 1 were recently molted adults, and A3 were adult individuals 1 week older than this. Line is the smoothed conditional mean with 95% confidence intervals.

**FIGURE 3:**
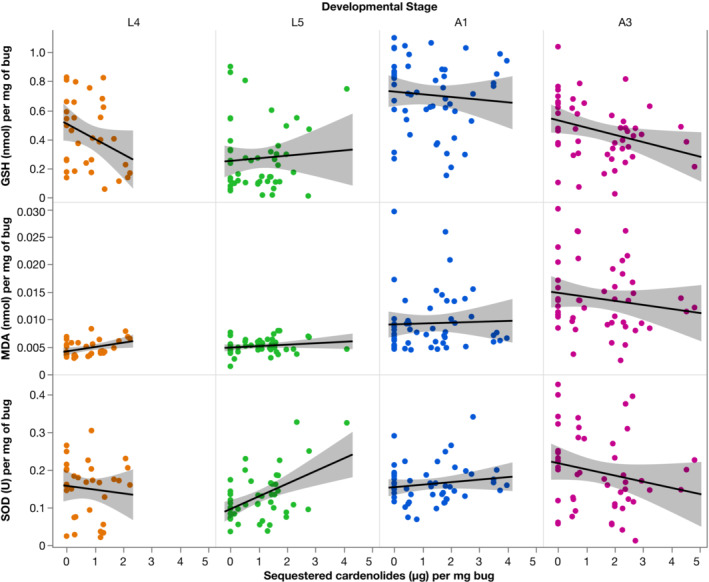
Correlation between sequestered cardenolides and oxidative stress markers in *O. fasciatus* individuals (*N* = 183). L4, L5 represent the nymphal stages Level 4 and 5. Adult 1 were recently molted adults, and A3 were adult individuals 1 week older than this. Left: total glutathione content (GSH); Middle: concentrations of MDA; Right: SOD activity. Line is the smoothed conditional mean with 95% confidence intervals.

Bugs that sequestered more cardenolides had a significantly lower levels of total glutathione content (GSH; Figure 2, *F*_1,157.4_ = 3.97, *p* = .048). GSH differed significantly between developmental stages (Figure 3, *F*_3,172.3_ = 31.017, *p* < .0001). L5 bugs had significantly lower levels of GSH than L4 (estimate = −0.19 ± 0.03), whereas A1 and A3 had higher levels of GSH than L4 (A1 estimate: 0.25 ± 0.03, A3 estimate: 0.01 ± 0.03). There was no significant interaction between cardenolide sequestration and developmental stages on the levels of GSH (*F*_3,172.1_ = 1.50, *p* = .21).

There was a significant interaction between cardenolide sequestration and developmental stages on SOD (Figure 3; *F*_3,173.1_ = 5.04, *p* = .002). A3 adult bugs had lower amounts of SOD activity with increasing sequestration compared to L4 bugs (estimate = −0.02 ± 0.008, *t* = −2.35, *p* = .02), and L5 bugs had significantly more SOD activity with increasing cardenolide sequestration compared to L4 bugs (estimate = 0.03 ± 0.009, *t* = 3.08, *p* = .002). There was a significant main effect of developmental stage on SOD activity (*F*_3,173.2_ = 7.19, *p* < .0001) with L5 bugs having lower SOD activity than L4 bugs (estimate = −0.03 ± 0.008, *t* = −2.91, *p* = .004), A3 bugs having significantly higher SOD activity than L4 bugs (estimate = 0.04 ± 0.008, *t* = 4.26, *p* < .0001). There was no significant difference in SOD activity between L4 and A1 bugs (estimate = −0.003 ± 0.008, *t* = −0.42, *p* = .68).

Cardenolide sequestration had no significant effect on MDA (*F*_1,174.7_ = 0.14, *p* = .71) but differed significantly between developmental stages (Figure 3, *F*_3,173.6_ = 40.1753, *p* < .0001). L5 bugs had lower levels of MDA than L4 (estimate = −0.003 ± 0.0006, *t* = −5.66, *p* < .0001), whereas A1 and A3 bugs had higher levels of MDA than L4 (A1 estimate = 0.0009 ± 0.0005, *t* = 1.73, *p* = .08; A3 estimate = 0.005 ± 0.0006, *t* = 9.96, *p* < .0001). There was no significant interaction between cardenolide sequestration and developmental stages (*F*_3,173.3_ = 0.99, *p* = .39).

### 3.3 Sequestration, oxidative stress, and warning signals

**FIGURE 4:**
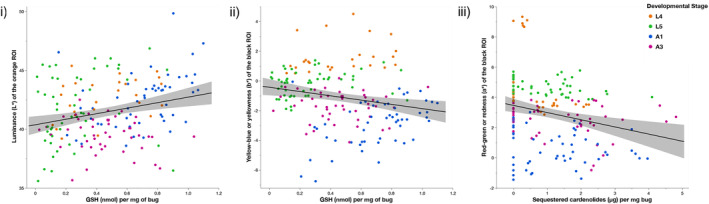
The relationship between total glutathione and sequestered cardenolides and warnings signals (i) GSH and luminance of the orange patches of *O. fasciatus* wings (*N* = 174) (ii) GSH and yellowness of the black patches, and (iii) sequestered cardenolides and redness of the black patches. Nymphal stages are L4 and L5, and A1 and A3 are adults. Line is the smoothed conditional mean with 95% confidence intervals.

Because GSH was the only marker associated with cardenolide sequestration, we proceeded to analyze its association with warning signals, but did not conduct analyses on the effects of MDA or SOD on warning signals (Figure 4).

#### 3.3.1 Brightness

We found a significant effect of GSH (Figure 4i; *F*_1,165.4_ = 10.45, *p* = .0015) and developmental stage (*F*_3,166_ = 17.39, *p* < .0001) on the brightness of the orange patches (luminance L*), but we did not find a significant effect of sequestered cardenolides on the orange patch brightness (*F*_1,166.5_ = 0.08, *p* = .77). We found no significant interaction between cardenolide sequestration and GSH on brightness (*F*_1,165.2_ = 0.55, *p* = .46). Bugs with higher amounts of GSH had brighter orange warning signals (estimate = 2.34 ± 0.72). L5 nymphs had significantly brighter orange patches than L4 (estimate = 0.67 ± 0.32, *t* = 2.11, *p* = .03), and A3 bugs had significantly less bright orange patches than L4 (estimate = −2.05 ± 0.28, *t* = −7.21, *p* < .0001). There was no significant difference in the brightness of the orange patches between L4 and A1 bugs (estimate = 0.32 ± 0.32, *t* = 0.98, *p* = .33).

There was a significant effect of developmental stage on the brightness of the black patches (*F*_3,167_, *p* = 348.26), but no significant effect of GSH (*F*_1,167_ = 0.69, *p* = .41) or cardenolide sequestration (*F*_1,167_ = 2.03, *p* = .16), and no significant interaction between cardenolides and GSH (*F*_1,167_ = 0.549, *p* = .46). L4 bugs had significantly less bright black patches than all other stages (L4–L5 estimate = 5.02 ± 0.48, *t* = 10.52, *p* < .0001; L4–A1 estimate = 6.28 ± 0.48, *t* = 13.13, *p* < .0001; L4–A3 estimate = 4.94 ± 0.43, *t* = 11.50, *p* < .0001).

#### 3.3.2 Red‐green chroma (a*)

We found a significant effect of developmental stage on the redness of the orange patches (a* values) (*F*_3,165.2_ = 352.14, *p* < .0001), but we did not find significant associations between GSH levels (*F*_1,166.4_ = 0.19, *p* = .66) or cardenolide sequestration on orange patch redness (*F*_1,163.7_ = 0.33, *p* = .57), and no significant interaction between cardenolides and GSH (*F*_1,165.9_ = 1.948, *p* = .16). A1 and A3 adults had significantly less red orange patches than L4 bugs (A1 estimate = −3.16 ± 0.26, *t* = −12.3, *p* < .0001; A3 estimate = −5.15 ± 0.26, *t* = −22.84, *p* < .0001) and there was no significant difference in orange patch redness between L4 and L5 bugs (estimate = −0.21 ± 0.25, *t* = −0.83, *p* = .41).

We found a significant effect of cardenolide sequestration on the redness of the black patches (Figure 4iii; *F*_1,167_ = 7.17, *p* = .008), and a significant effect of developmental stage (*F*_3,167_ = 80.43, *p* < .0001). We found no significant effect of GSH (*F*_1,167_ = 2.67, *p* = .10) and no significant interaction between cardenolide and GSH (*F*_1,167_ = 0.26, *p* = .61). Bugs that sequestered more cardenolides had less red black patches (estimate = −0.22 ± 0.24). L5 bugs had significantly redder black patches than L4 (estimate = 1.58 ± 0.19, *t* = 8.51, *p* < .0001), and adult A1 and A3 had significantly less red black patches than L4 (A1 estimate = −2.60 ± 0.19, *t* = −13.96, *p* < .0001; A3 estimate = −0.64 ± 0.17, *t* = −3.84, *p* = .0002).

#### 3.3.3 Yellow‐blue chroma (b*)

We also found a significant effect of developmental stage on the yellowness of the orange patches (b* values) (*F*_3,166_ = 57.51, *p* < .0001), but no significant effect of GSH (*F*_1,165.4_ = 1.43, *p* = .23) or cardenolide sequestration (*F*_1,166.6_ = 0.03, *p* = .86), and no interaction between GSH and cardenolides on redness (*F*_1165.2_ = 1.34, *p* = .25). L4 bugs were significantly more yellow than all other developmental stages (L4–L5, estimate = −2.12 ± 0.38, *t* = −5.53, *p* < .0001; L4–A1, estimate = −1.91 ± 0.39, *t* = −4.33, *p* < .0001; L4–A3, estimate = −1.63 ± 0.34, *t* = −4.75, *p* < .0001).

We found a significant effect of GSH (Figure 4ii; *F*_1,166.2_ = 11.75, *p* = .0008) and developmental stage (*F*_3,165_ = 98.99, *p* < .0001) on the yellowness of the black patches. We found no significant effect of cardenolide sequestration (*F*_1,164.1_ = 0.20, *p* = .65) and no significant interaction between cardenolide sequestration and GSH (*F*_1,165.6_ = 0.20, *p* = .65). Bugs with higher levels of GSH had yellower black patches (estimate = 1.29 ± 0.38, *t* = 3.43, *p* = .0008). L5 bugs had significantly yellower black patches than L4 (estimate = 0.91 ± 0.16, *t* = 5.59, *p* < .0001), and adult A1 and A3 bugs had significantly less yellow black patches than L4 bugs (A1 estimate = −2.41 ± 0.17, *t* = −14.36, *p* < .0001; A3 estimate = −0.91 ± 0.15, *t* = −6.20, *p* < .0001).

## 4 DISCUSSION

We reared large milkweed bugs *O. fasciatus* on an artificial diet with increasing concentrations of cardenolides and found intra‐individual variation in sequestration. Sequestration of cardenolides was associated with a depletion of the antioxidant defense total glutathione (GSH), and reduced levels of GSH were associated with decreased luminance of the orange body/wing patches, and decreased chroma (blueness) of the black hemelytra patches. Our results provide further evidence that sequestration of secondary metabolites is costly for aposematic animals (Agrawal et al., 2021; Lindstedt et al., 2010; Reudler et al., 2015), and that sequestration of secondary metabolites can impact oxidative state (Ahmad, 1992; Blount et al., 2009, 2023; Tollrian & Harvell, 1999). Milkweed bugs that sequestered more cardenolides also had lower redness of the black hemelytra patches compared to bugs that sequestered less cardenolides. This is in line with research showing that resource availability can influence the expression of melanic signals in other aposematic species (Lindstedt et al., 2020). Investigating how resource availability affects the defenses of aposematic animals is important for understanding which factors drive variation in warnings signals and chemical defenses (Barzaghi et al., 2022; Blount et al., 2009, 2012; Davis, 2009; Lindstedt et al., 2020; Ottocento et al., 2023).

If pigments used in prey warning signals play a dual role in producing both the signal and in preventing self‐damage when storing toxins (due to their antioxidant properties; Blount et al., 2009), we would expect an interaction between sequestration and oxidative state on signaling if bugs are signaling honestly. We did not find an interaction for any color metrics. Instead we found that when *O. fasciatus* were raised on higher concentrations of cardenolides they had lower levels of total glutathione, which suggests that sequestration depletes this antioxidant. Glutathione is involved in detoxification processes (Enayati et al., 2005), and is well‐known for detoxifying phytochemicals such as aristolochic acid (Gao et al., 2021) and sulforaphane (Villa‐Cruz et al., 2009). Glutathione is also involved in the melanin synthesis pathway when pheomelanin dopaminquinone undergoes a non‐enzymatic conjugation of a thiol, usually glutathione or cysteine to produce thiolated catecholamines (Ito & Prota, 1977). Although the orange pigments in large milkweed bugs have been identified as pterins (Bartel et al., 1958), pteridines also commonly act as cofactors of enzymes involved in the melanin synthesis pathway which hydroxylate phenylalanine to tyrosine and oxidize tyrosine to DOPA (Shamim et al., 2014). Given that glutathione was depleted with increasing concentrations of sequestered cardenolides, and individuals that had low levels of GSH produced less bright orange warning signals, and that the chroma of the melanin‐based signals were correlated with sequestration, we can speculate that glutathione availability has a role in the biochemistry underlying the variation in colouration. GSH also varied across developmental stage. How GSH varies during feeding, molting, and growth across generations, and how this affects adult signaling in the long‐term, warrants further study. Pigmentation and warning colors are regulated by more than one mechanism, and our results show that the relationship is likely more complex than our study is able to show, and that this warrants further psychophysical and biochemical investigation.

Our results could also reflect differences in how we quantified the visual signals of the bugs. In this study we calculated hue and luminance according to trichromatic L*a*b* color space rather than Δ*S* conspicuous to a specific background for a mono, di, or tetrachromatic visual system (Troscianko & Stevens, 2015). Modeling appearance for a range of visual systems and natural backgrounds, as well as calculating the internal contrast of the two color components of the signals, is beyond the scope of the present study but is advised in future research on milkweed bug color. It is possible that the variance in the hue of the black hemelytra patches that correlated with sequestration would be difficult to detect because the Euclidian distance varies by ~2.5, and because of the geometry of Lab color space where the length of the b* axes is reduced for dark colors. Whether the resource‐dependent variation in warning signals and cardenolide concentrations are perceived by natural predators will be important to test in the future to understand how variation and correlations in primary and secondary defenses is maintained in aposematic species (Ottocento et al., 2023; Petschenka et al., 2022; Pokharel et al., 2020; White & Umbers, 2021).

We predicted a positive correlation between individual levels of cardenolides sequestered by *O. fasciatus* and oxidative lipid damage. Instead we found stable levels of oxidative damage (MDA) during sequestration but changes in antioxidant defenses. This suggests that *O. fasciatus* sustain redox state during acquisition of cardenolides by using antioxidant defenses. In a comparator system, the monarch butterfly (*Danaus plexippus*), increasing concentrations of sequestered cardenolides resulted in increased oxidative damage (Blount et al., 2023). *O. fasciatus* have significantly higher resistance to cardenolides than monarchs (Bramer et al., 2015), which could be one explanation for the difference in our results. Another possibility is that it is due to our holding the nutritional background constant, only varying the quantity of additional cardenolides, whereas in Blount et al. (2023) monarchs were reared on whole food plants which differ not only in phytochemical profile but also in other metabolic and physical parameters that could have contributed further to changes in redox state. We also found that *O. fasciatus* only sequestered ouabain, and there were no digitoxin metabolites (as was also reported in Pokharel et al., 2021), so the costs of sequestration may differ when milkweed bugs are feeding on plants with more complex mixtures of cardenolides that require metabolic transformation (see also Agrawal et al., 2022). *O. fasciatus* do experience oxidative damage in other contexts (López‐Muñoz et al., 2019), so the stable levels of damage we measured here could be related to their higher cardenolide resistance rather than a general resistance to oxidative stress. A comparative analysis of related milkweed bug responses to sequestration would be worthwhile, and aid in our understanding of host shifts that are known to occur in the Lygaeinae (Petschenka et al., 2022).

We controlled for batch effects in our results. These batch effects could be due to variation in the length of exposure to toxins during the bugs' development. However, we checked all batches and insect boxes daily to monitor their molting and sampled them according to the same criteria across batches. Another reason could be genetic variance among batches, which has been described in studies on flight (Palmer & Dingle, 1986, 1989) and heritability of morphological traits (Rodríguez‐Clark, 2004). Our bugs, however, came from the same long‐term laboratory colony. It could also be argued that our experimental design caused these batch effects because we have a single factor treatment with four levels (diet) randomly assigned to an experimental box in each batch. This means that we potentially introduced a unique set of factors to each box and batch that contribute to the error variance of the measures of the response in that group and, as a consequence, the error (residuals) within a batch are more similar to each other than they are to the residuals among batches. Our batch effects were mainly at the level of sequestration rather than across all measures of oxidative state and colouration, and this could reflect intra‐individual variation in sequestering efficiency which has been recorded in wild caught populations (Isman et al., 1977). Irrespective of these limitations, our results demonstrate that the amount of cardenolides sequestered can influence the redox state of large milkweed bugs, and that antioxidant availability affects warning signal brightness and chroma.

While the overall tolerance of large milkweed bugs to cardenolides is well established (Bramer et al., 2015; Isman, 1977; Lohr et al., 2017; Scudder et al., 1986), our results add to the recent evidence that sequestration of cardenolides by *O. fasciatus* impacts life‐history traits (Agrawal et al., 2022; Pokharel et al., 2021), and has molecular costs (Dalla et al., 2017; Dalla & Dobler, 2016). This is after what Agrawal et al. (2022) describe as “a long history of finding no costs of cardenolide exposure or sequestration” (Chaplin & Chaplin, 1981; Isman, 1977; Vaughan, 1979). Our results also add to the growing literature that the production and maintenance of warning signals and chemical defenses are affected and limited by resource availability (Barzaghi et al., 2022; Blount et al., 2009, 2012, 2023; Burdfield‐Steel et al., 2019; Lindstedt et al., 2010, 2020; Ottocento et al., 2023).

## AUTHOR CONTRIBUTIONS

**H. Cecilia Heyworth:** Data curation (lead); formal analysis (lead); investigation (lead); methodology (equal); writing – original draft (lead). **Prayan Pokharel:** Data curation (lead); formal analysis (lead); investigation (lead); methodology (equal); visualization (lead); writing – original draft (lead). **Jonathan D. Blount:** Conceptualization (lead); methodology (equal); resources (equal); supervision (lead); writing – review and editing (equal). **Christopher Mitchell:** Methodology (equal); validation (equal); writing – review and editing (equal). **Georg Petschenka:** Funding acquisition (lead); Damethodology (equal); resources (equal); supervision (lead); writing – review and editing (equal). **Hannah M. Rowland:** Conceptualization (lead); data curation (equal); formal analysis (equal); funding acquisition (lead); methodology (equal); project administration (lead); resources (equal); supervision (lead); validation (equal); writing – original draft (equal).

## FUNDING INFORMATION

This research was funded by the Max Planck Society funding to HMR, and the LOEWE program of the State of Hesse via funding the LOEWE Center for Insect Biotechnology & Bioresources, and DFG grant PE 2059/3‐1 to GP. HCH was supported by an International Max Planck Research School fellowship.

## CONFLICT OF INTEREST STATEMENT

The authors have no conflicts of interest.

## Supporting information


Data S1

Figures S1–S3

## Data Availability

The data on color, oxidative state (MDA, GSH, and SOD), and cardenolide concentration is available in the Supplementary Material.
