# Supplementary material for: Antioxidant availability trades off with warning signals and toxin sequestration in the large milkweed bug (Oncopeltus fasciatus)
Source: Ecol Evol. 2023 Apr 7;13(4):e9971. doi: 10.1002/ece3.9971 (PMC10082154; doi:10.1002/ece3.9971)
Supplement: Supplementary file 2 — Figures S1–S3 [file ECE3-13-e9971-s001.docx]

**Supplementary Material**

**Antioxidant availability trades off with warning signals and sequestration in the large milkweed bug (*Oncopeltus fasciatus*)**

**Heyworth, H.C.^1,3,*^, Pokharel, P.^2,*^, Blount, J.D.^3^, Mitchell, C.^3^, Petschenka, G.^2^, Rowland, H.M.^1^**

**Contents**

1. **Figure S1.** Amount of cardenolides in the artificial diet. Control, Low, Medium, and High diets had 0 mg/g, 2 mg/g, 6 mg/g, 10 mg/g equimolar ouabain and digitoxin added respectively.
2. **Figure S2**. Reflectance spectra generated from the filter apparatus LEDs.
3. **Figure S3.** Regions of Interest selected in the different larval stages of *Oncopeltus fasciatus*. Yellow areas were the red selections, and blue were the black, with areas of glare removed.

**1.**


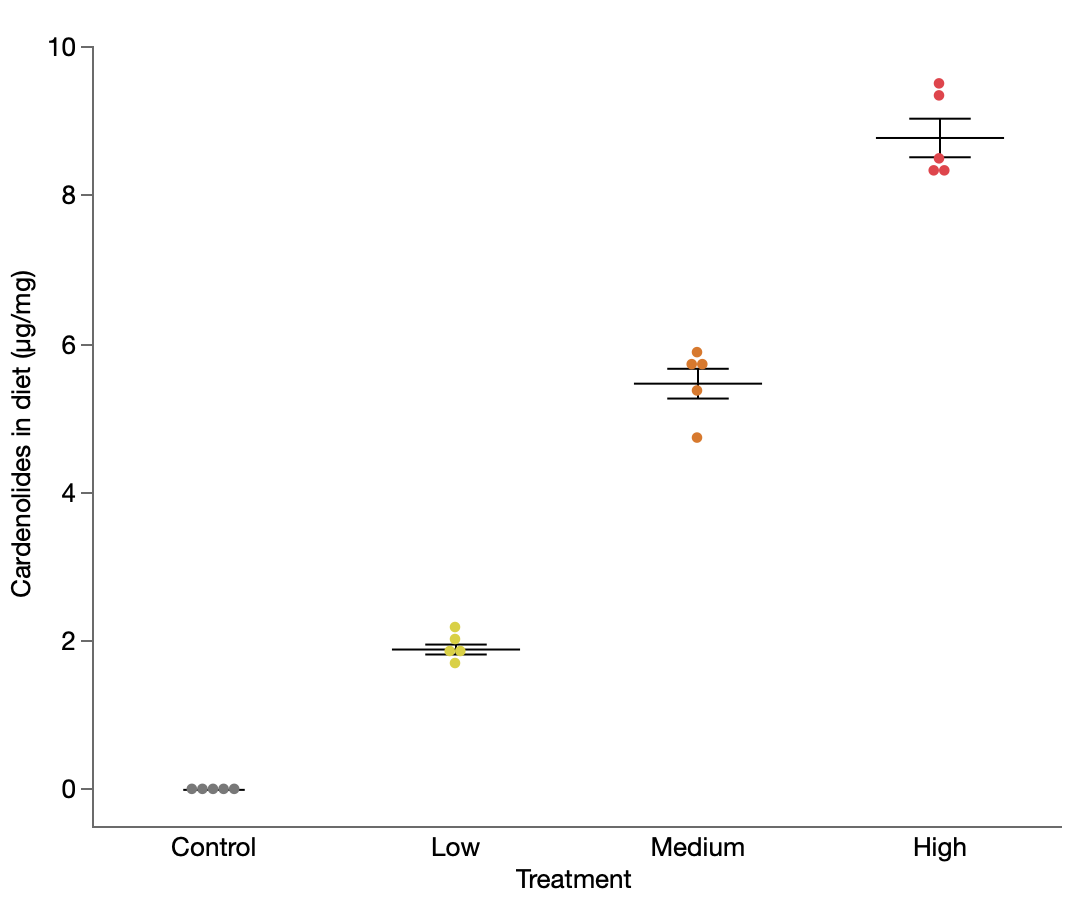


**Figure S1.** Amount of cardenolides in the artificial diet. Control, Low, Medium, and High diets had 0 mg/g, 2 mg/g, 6 mg/g, 10 mg/g equimolar ouabain and digitoxin added respectively.

**2.**


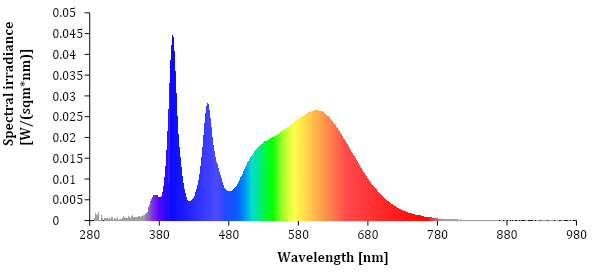


**Figure S2**. Reflectance spectra generated from the filter apparatus LEDs.

3.
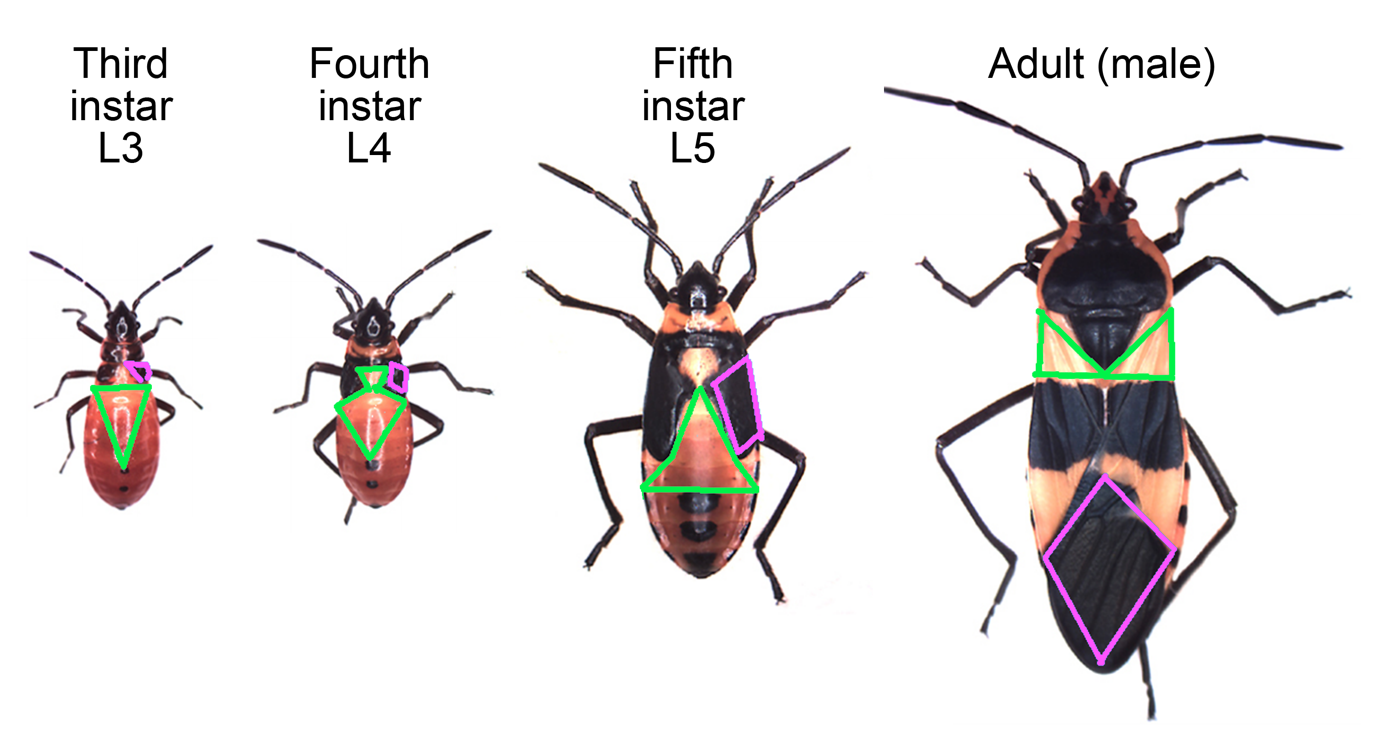


**Figure S3.** Regions of Interest selected in the different larval stages of *Oncopeltus fasciatus*.
